# Sarcopenia: Body Composition and Gait Analysis

**DOI:** 10.3389/fnagi.2022.909551

**Published:** 2022-07-13

**Authors:** Yuxuan Fan, Bo Zhang, Guohao Huang, Guoying Zhang, Zhiyuan Ding, Zhiyu Li, Jonathan Sinclair, Yifang Fan

**Affiliations:** ^1^Foot Research Laboratory, School of Physical Education and Sport Science, Fujian Normal University, Fuzhou, China; ^2^College of Sports and Health, Guangzhou Sport University, Guangzhou, China; ^3^College of Foreign Studies, Jinan University, Guangzhou, China; ^4^Research Centre for Applied Sport, Physical Activity and Performance, School of Sport and Health Sciences, University of Central Lancashire, Preston, United Kingdom

**Keywords:** sarcopenia, body composition, gait parameters, butterfly parameters, gait forward dynamics method

## Abstract

**Background:**

Age-induced sarcopenia negatively affects walking stability and increases the risk of falls, which is the leading cause of accidental death in the elderly.

**Objective:**

This study aimed to analyze and contrast body composition and gait characteristics in those with sarcopenia in relation to healthy controls to shed some light on the prevention of falls in elderly patients with sarcopenia.

**Materials and Methods:**

In this study, 68 community dwellers were scanned by the Hologic QDR-4500A Dual-energy X-ray absorptiometry (DXA). The appendicular lean mass index (ALMI) results were used to distinguish the normal participants from those with sarcopenia: 24 in the sarcopenia group, and 44 into the normal group. The participants were asked to undergo gait analysis on a plantar pressure measurement system. Statistical analysis was conducted to contrast both groups' gait and butterfly parameters from their gait test, and then a gait forward dynamics method was performed to quantify the analysis for both groups.

**Results:**

The ALMI of the female was not related to their age (*r* = 0.06) while that of the male was weakly related (*r* = 0.17). Body mass index (BMI) from both groups was normal, although with a statistically greater BMI from the normal group compared with sarcopenia (*p* < 0.001). Greater values and significant differences were found in step length and stride length from the normal elderly group (*p* < 0.01), and so was the length of the gait line and single support line (*p* < 0.05). Gait forward dynamics analysis results showed no motor neural or musculoskeletal disorders in their gait performance from the sarcopenia group.

**Conclusion:**

For the elderly, age did not largely affect the ALMI, BMI, or *T-*score, but BMI and ALMI were strongly correlated. In this study, significant differences were found in certain gait parameters between the elderly with sarcopenia and the normal elderly, which were related to absolute muscle strength, suggesting that sarcopenia was a disease mainly caused by decreased muscle mass. In addition, when abnormities were identified in step length, stride length, length of gait line, or length of single support line, it is proposed to take a DXA scan to confirm whether the elderly suffer from sarcopenia.

## Introduction

Over the past few decades, the decrease in fertility rate and the increase of life expectancy (Li and Lin, 2016) have caused China to be one of the fastest population aging countries in the world (Phillips and Feng, 2015). Aging is one of the major challenges for Chinese society (Han et al., 2020), leading to body structure senescence and motor function decline. The lower limb muscles in the elderly have a reduced cross-sectional area by 25–35% compared with those of the young (Lexell, 1995), explaining why the elderly are more vulnerable to sarcopenia. Sarcopenia is characterized by symptoms of age-related loss of skeletal muscle mass and poor muscle strength or physical fitness (Cruz-Jentoft et al., 2010; Kirk et al., 2021). Differences in age, gender, and race mediate different degrees of muscle loss. Among the elderly over 65 years of age, the age- and sex-adjusted prevalence of sarcopenia was 6–15%, depending on the assessment of muscle mass parameters (Baumgartner et al., 1998; Iii et al., 2000).

Sarcopenia is a complex syndrome caused by many factors (Ribeiro and Kehayias, 2014). The quality of lean principle proposes to utilize body composition assessment to indirectly assess sarcopenia. This principle connects muscle mass and metabolic function to fat-free mass (Ribeiro and Kehayias, 2014). Research findings have shown that sarcopenia presents in an impaired state of health, and is related to falls, bone fracture risk, movement disorders, reduction of daily activities, disabilities, loss of independence, and an increased risk of death (Cawthon et al., 2007; Cruz-Jentoft et al., 2010; Morley et al., 2011; Fukuoka et al., 2019; Coll et al., 2021). For instance, the decrease in walking stability and the increased risk of falling might lead to the loss of physical function independence (Dutta, 1997). Other consequences of sarcopenia included the increased risks of chronic diseases, such as diabetes and osteoporosis (Dutta, 1997; Fukuoka et al., 2019).

The clinical diagnosing standard of sarcopenia by the European Working Group on Sarcopenia in Older People (EWGSOP) included low muscle mass, low muscle strength, or poor physical performance (Cruz-Jentoft et al., 2010). The Asian Working Group for Sarcopenia (AWGS) issued regional consensus guidelines in 2014, which were mostly consistent with those from EWGSOP (Chen et al., 2014). Clinically assessment techniques included bioelectrical impedance analysis (BIA), dual energy X-ray absorptiometry (DXA), and anthropometric measures to calculate muscle mass (Falsarella et al., 2014).

To avoid suffering from sarcopenia, walking speed was one of the most frequently used measurements to assess the elderly's ability to live independently (Kang and Dingwell, 2008; Bohannon and Andrews, 2011), because muscle mass was closely associated with motor function (Rosenberg, 1997). Other syndromes or diseases might affect the accuracy of using walking speed to assess muscle function, though. In addition, the decreased hip movements in the sagittal plane and the increase in pelvic tilt in the anteroposterior plane extended the support phase and reduced the stride time (Sanchez-Rodriguez et al., 2015), which may, at the same time, affect the accuracy of using walking speed as an assessment technique. A comprehensive gait analysis served as a useful tool to assess muscle loss, whereas cameras and force plates were gold-standard tools to assess gait clinically (Kim et al., 2021). Gait analysis could be used to predict the risk of fall, or to distinguish age groups, disease types, or physical activity levels (Senden et al., 2009; Bautmans et al., 2011), to identify abnormal gait characteristics and to provide additional information about the patients' degree of functionality or their risk of fall (Senden et al., 2012; Thiede et al., 2016). To assess and identify factors that may impair gait stability was essential to design intervention programs to maintain the independence and mobility of the elderly (Leiros-Rodriguez et al., 2018).

This study aimed to explore body composition and gait characteristics in those with sarcopenia in relation to healthy controls as well as the relationship among body composition, gait parameters, and sarcopenia to shed some light on the prevention of falls among the elderly.

## Materials and Methods

### Participants

Community-dwelling elderly (aged 53–78 years) participants were recruited—their body composition was measured by a DXA scanner (Hologic QDR-4500A). Appendicular lean mass (ALM) was calculated by collecting the sum of arm and leg lean mass. Based on their appendicular lean mass [ALMI, height, H; ALMI = ALM/H^2^ (kg/m^2^)], the sarcopenia group (*n* = 24) was identified, i.e., ALMI < 7.0 kg/m^2^ in men and 5.8 kg/m^2^ in women (Tanimoto et al., 2014). Those without low muscle mass or strength and low physical performance were classified into the normal group (*n* = 44). Participants' basic information is shown in Table 1.

**Table 1:** Basic information of the participants.

	**Sarcopenia (*****n*** **=** **24)**		**Normal (*****n*** **=** **44)**	
	**Male (*n* = 5)**	**Female (*n* = 19)**	**Male (*n* = 26)**	**Female (*n* = 18)**
Age, year	66.60 ± 8.17	62.68 ± 6.01	64.58 ± 5.22	63.61 ± 6.99
Height, m	161.52 ± 6.55	156.64 ± 6.14	169.10 ± 4.03	157.78 ± 4.43
Weight, kg	56.44 ± 7.27	52.68 ± 5.58	72.29 ± 7.94	59.92 ± 5.98
BMI^*^	21.57 ± 1.79	21.46 ± 1.77	25.26 ± 2.46	24.07 ± 2.27
ALM^#^, g	16193.22 ± 2534.52	13369.89 ± 1553.64	23226.45 ± 2008.18	15731.66 ± 1392.78
ALMI^@^ kg/m^2^	6.18 ± 0.58	5.43 ± 0.31	8.12 ± 0.62	6.32 ± 0.44
*T*-score	−1.74 ± 1.59	−1.72 ± 1.30	−0.21 ± 1.04	−0.83 ± 1.12

* *BMI, body mass index*;

# *ALM, appendicular lean mass index*;

@ *ALMI [H, height, ALMI=ALM/H^2^(kg/m^2^)]*.

All participants' annual medical reports were checked to exclude patients with neural or musculoskeletal disease. The experimental procedures were explained to the participants and written informed consent forms were obtained from the participants before testing. This study was approved by the Ethics Committee of Fujian Normal University.

### Equipment

**Figure 1:**
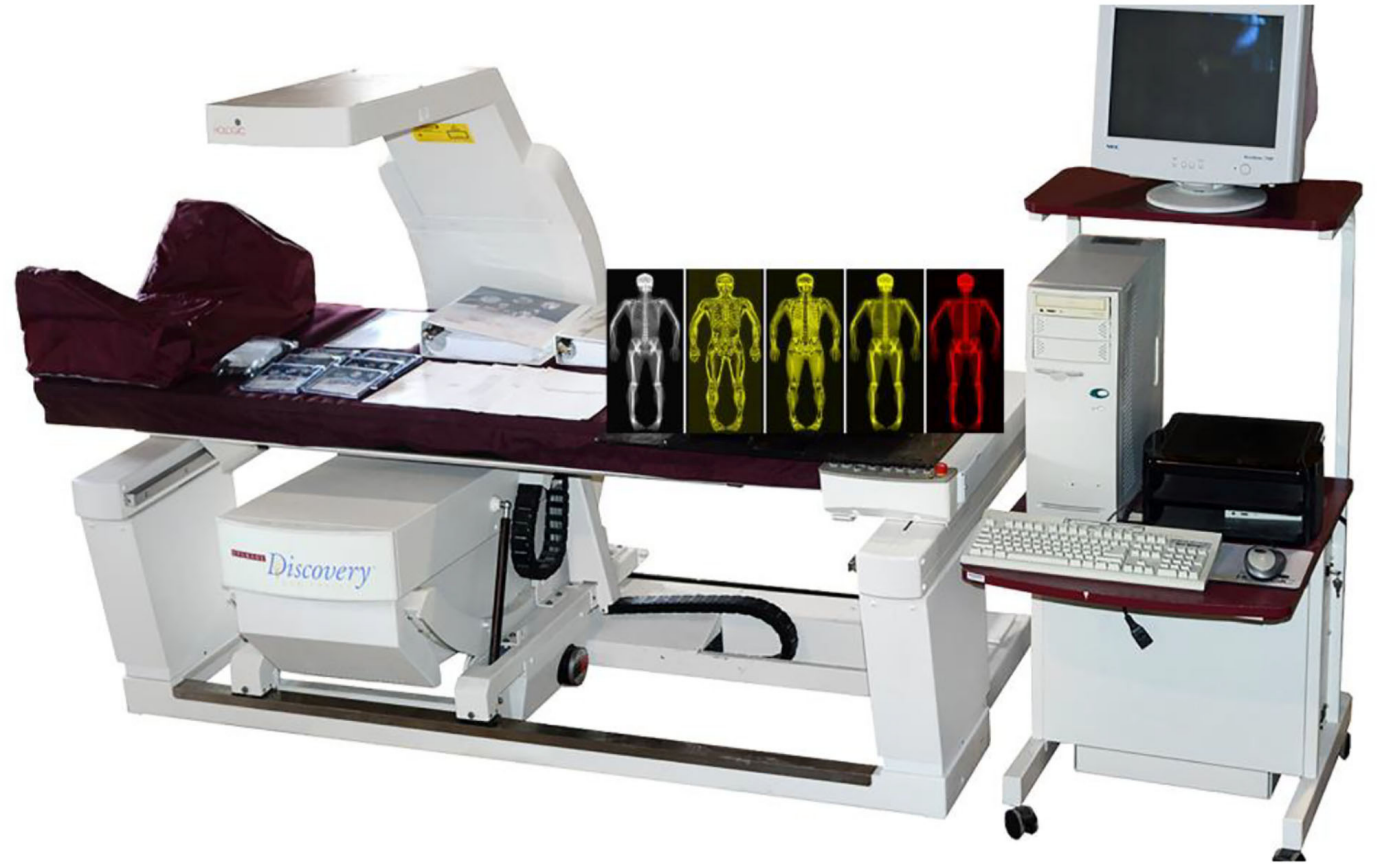
A dual-energy X-ray absorptiometry (DXA) scanner (Hologic QDR-4500A).

A bone mineral density scanner DXA system (Hologic QDR-4500A; Hologic, Mass, USA) was utilized. The system was allowed to warm up for 10 min prior to use, and self-tests were undertaken on a daily basis in accordance with the manufacturer's requirements. The scanning parameters were as follows: 140/100 kVp, 2.5 mA; scanning mode: e whole body; software: Version 12.4:3; analysis: Auto Whole Body. Figure 1 shows the equipment.

**Figure 2:**
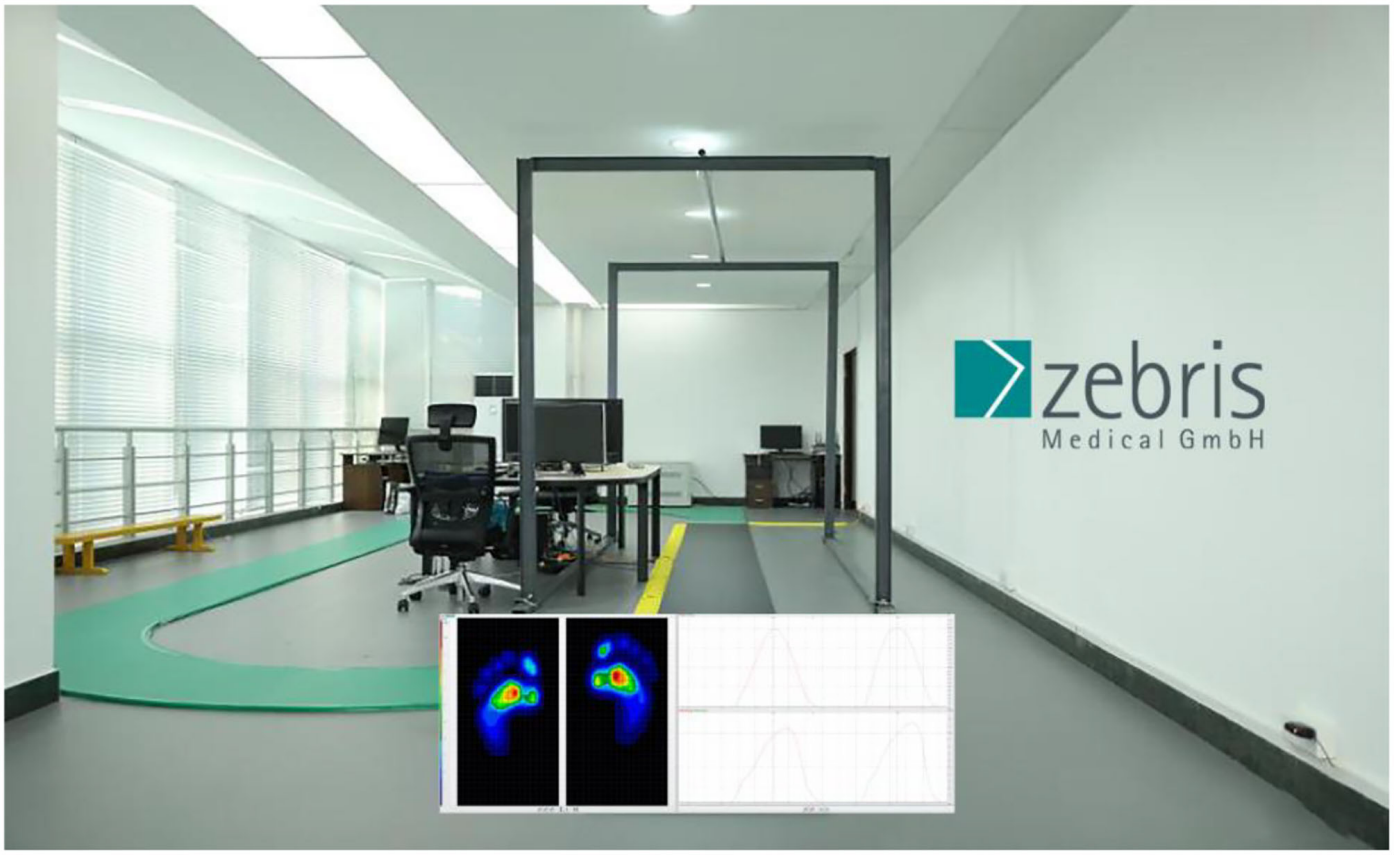
A Zebris FDM plantar pressure measurement system.

A Zebris FDM plantar pressure measurement system (Zebris Medical GmbH, Isny, Germany-6.08 (L) × 0.56 (W) m), with 45,056 sensors frequency of 100 Hz) was utilized. The system was calibrated prior to each testing session to ensure data integrity. Figure 2 shows the equipment.

### Gait Test and Calculation

Participants were asked to walk at a self-selected velocity on the Zebris FDM System. Data were collected at a sampling rate of 100 Hz. For each participant, data that included 10 complete successive foot contacts were accepted and then analyzed. Gait parameters were processed by the Win FDM (V1.18.40).

### Gait Forward Dynamics Method

For bipedal walking, the ground reaction force (GRF) satisfies the following conditions:


(1) $$\begin{array}{c} \int_{0}^{T}(F_{1}(t)+F_{2}(t)-mg)dt=0 \end{array}$$

where, *F*_1_(*t*) and *F*_2_(*t*) refer to the GRF of the left and right foot at a certain time, respectively; *T* the stride time, *t* a certain moment in a stride cycle, *m* the body mass, and *g* the acceleration of gravity.

Introduce the foot's ground contact moment *t*_*initial*_, *F*(*t* + *t*_*initial*_) GRF. Since *F*_1_(*t*) + *F*_2_(*t*) = *F*(*t* + *t*_*initial*_), the component form of GRF is as follows:


(2) $$\begin{array}{r} F\left(t+t_{initial}\right)=F_{x}\left(t+t_{initial}\right)+F_{y}\left(t+t_{initial}\right)+F_{z}\left(t+t_{initial}\right) \end{array}$$

where, *F*_*x*_(*t* + *t*_*initial*_), *F*_*y*_(*t* + *t*_*initial*_), *and F*_*z*_(*t* + *t*_*initial*_) refer to the components of GRF in *x, y, z* directions of the Cartesian coordinate system respectively, and *t* a certain moment in a stride cycle.

By Equations (1) and (2), restraint conditions of $\int_{0}^{T}F_{x}(t)dt=0$ in *x* direction will be


(3) $$\begin{array}{c} \int_{0}^{T}F_{x}(t)dt=\int_{0}^{T}F_{x}(t+t_{initial})dt=0 \end{array}$$

By Equations (1) and (2), restraint conditions of $\int_{0}^{T}F_{y}(t)dt=0$ in *y* direction will be


(4) $$\int_{0}^{T}F_{y}(t)dt=-\int_{0}^{T}F_{y}(t+t_{initial})dt$$

By Equations (1) and (2), restraint conditions of $\int_{0}^{T}F_{z}(t)dt=\int_{0}^{T}mgdt$ in *z* direction, and since $\int_{0}^{T}F_{z}(t)dt=\int_{0}^{T}(t+t_{o})dt$, we will get


(5) $$\int_{0}^{T}F_{z}(t)dt\begin{array}{c} \begin{array}{c} +\int_{0}^{T}F_{z}(t+t_{initial})dt=\int_{0}^{T}mgdt \end{array} \end{array}$$

By Equation (5), and by Newton's second law, the acceleration equation of the body's center of mass (COM) at a certain moment in a stride cycle in *z* direction will be


(6) $$\begin{array}{r} a_{z}\left(t+t_{initial}\right)=F_{z}\left(t\right)+F_{z}\left(t+t_{initial}\right)-mg \end{array}$$

where, *a*_*z*_ stands for acceleration, *F*_*z*_ GRF, *t* a certain moment in a stride cycle, *t*_*initial*_ initial ground contact moment, *m* body weight, and *g* the acceleration of gravity.

Take the body coordinates of COM as a non-inertial reference frame, and set the initial velocity of COM at the beginning of the stride cycle to be *v*_0_(*t*_*initial*_). By Equation (6), when the ground contact moment is *t*_*initial*_, the relationship between the centroid velocity at any time *t* in a stride cycle and the acceleration and initial velocity will be


(7) $$v(t,t_{initial})=\int_{0}^{t}a(t,t_{initial})dt+v_{0}(t_{initial})$$

*v*_*x*_, *v*_*y*_ and *v*_*z*_ stand for the velocities in the three directions of COM, respectively. So, $v_{x}(t,t_{initial})=\int_{0}^{t}a_{x}(t,t_{initial})dt+v_{x0}(t_{initial}),v_{y}(t,t_{initial})=\int_{0}^{t}a_{y}(t,t_{initial})dt+v_{y0}(t_{initial}),\;\;and\;v_{z}(t,t_{initial})=\int_{0}^{t}a_{z}(t,t_{initial})dt+v_{z0}(t_{initial})$. It seems impossible to determine the magnitude of *v*_0_(*t*_*initial*_) in Equation (7) by means of dynamics method, but based on the least-action principle in gait (Fan et al., 2009), *v*_0_(*t*_*initial*_) has a unique value. Equation (7) shows that *v*_0_(*t*_*initial*_) is a function of *t*_*initial*_ for the ground contact moment. The relationship between the initial velocity of relative motion and acceleration is as follows:


(8) $$v_{0}(t_{initial})=-\sum_{\lambda =1}^{T}\int_{0}^{\lambda }a(t,t_{initial})dt$$

For any ground contact moment, $\int_{0}^{T}a(t,t_{initial})dt=0$. By Equation (7), *v*(*T, t*_*initial*_) = *v*_0_(*t*_*initial*_). It should be noted that the initial velocity calculated by Equation (8) refers to the stable stepping speed of body at the beginning of the stride cycle, and it is the initial velocity of the COM relative to the dynamic reference frame, not the absolute velocity of the COM relative to the static reference frame. By Equations (6) and (8), the foot's ground contact moment determines the initial speed of relative motion, but it has nothing to do with the implicated velocity, which is quite intriguing. When running on a linear path, the commonly used gait parameter of “step velocity” is the implicated speed, and the step velocity is related to stride frequency and stride length. Relative velocity, therefore, is independent of stride frequency and stride length.

When the body coordinate of COM is used as a non-inertial reference frame, the sum of the potential energy of COM is $\overset{T}{\underset{1}{\sum }}E_{p}\left(t\right)=0$, and the mean potential energy is ${\bar{E}}_{p}=0$; the kinetic energy of COM *t* at any time of relative motion is *E*_*k*_(*t*)≥ 0. In a stride cycle, the sum of kinetic energy is $\overset{T}{\underset{1}{\sum }}E_{k}\left(t\right)>0$, and the mean kinetic energy is ${\bar{E}}_{k}>0$. Therefore, we use the relative motion centroid energy to simplify the description of mechanical energy consumption in gait as follows:


(9) $$\begin{array}{r} E\left(t,t_{initial}\right)=\frac{1}{2}v^{2}\left(t,t_{initial}\right) \end{array}$$

Vertical ground reaction force (VGRF) and gravity from Zebris FDM System gait analysis report were normalized by self-weight, and stride cycle normalized by percentage. Equations (1)–(9) were established to calculate both groups' VGRF, acceleration, velocity, displacement, and dynamic energy of COM, respectively. An application of these equations is shown in Supplementary Tables 1–3.

### Statistical Analyses

Data from the sarcopenia and normal groups were expressed in the format of means ± standard deviation (SD). Gait parameters from the two groups were compared using an independent sample *t*-test analysis (two-tailed distribution). In addition, linear associations between the experimental measurements were explored using correlation analyses. Correlation analyses were interpreted as follows: 0–0.2 = very weak, 0.21–0.4 = weak, 0.41–0.6 = moderate, 0.61–0.8 = strong, and 0.81–1.0 = very strong. The statistical significance level throughout was determined at the *p* ≤ 0.05 level.

## Results

### Correlations

**Figure 3:**
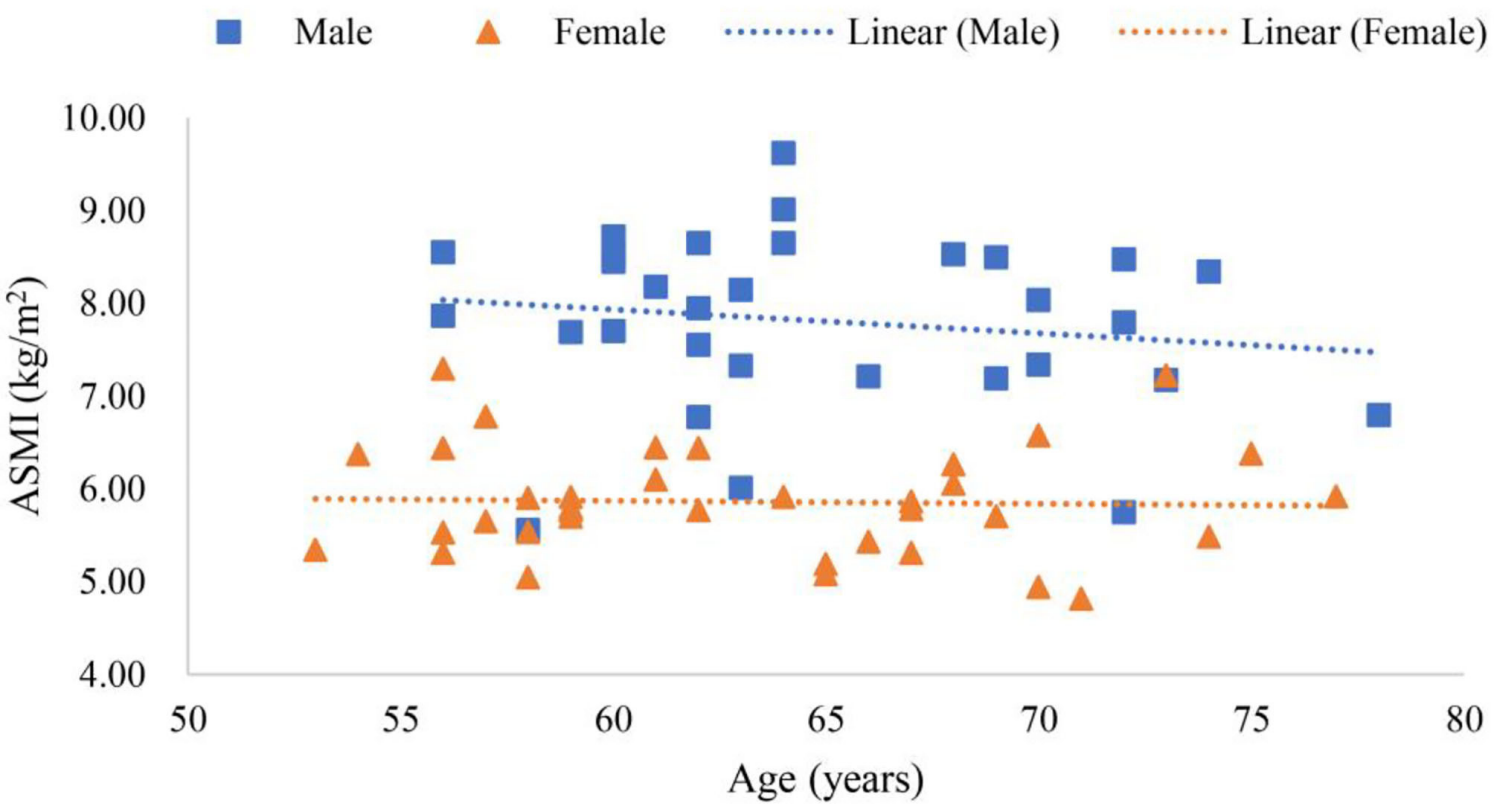
Scatter plots of participants' age and appendicular lean mass index (ALMI).

**Figure 4:**
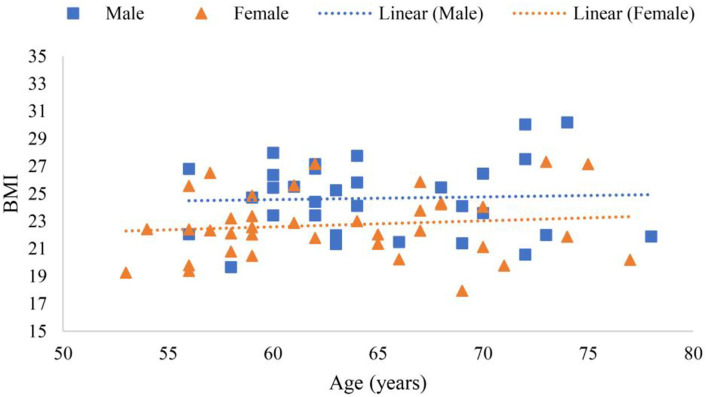
Scatter plots of participants' age and body mass index (BMI).

**Figure 5:**
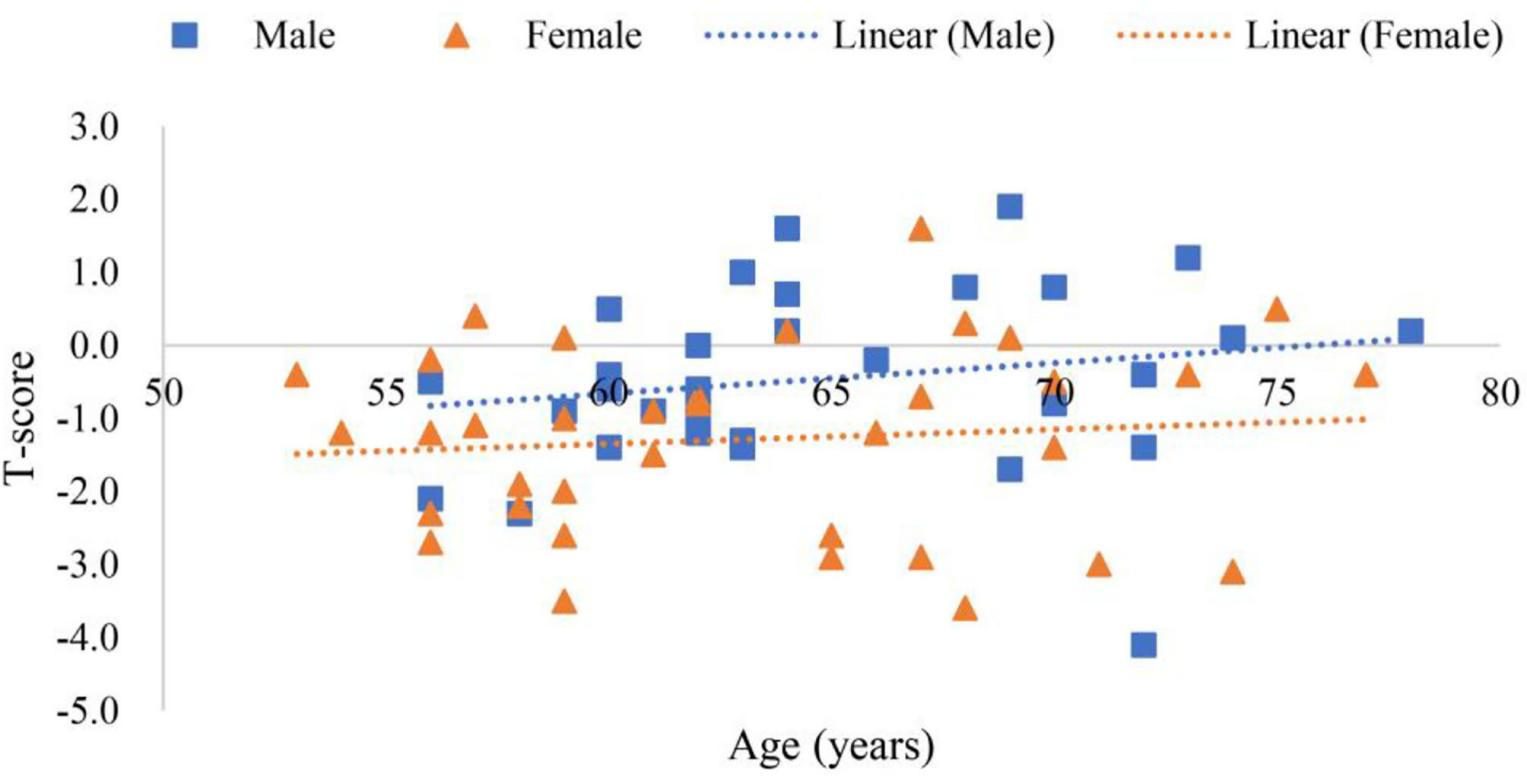
Scatter plots of participants' age and *T*-score.

**Figure 6:**
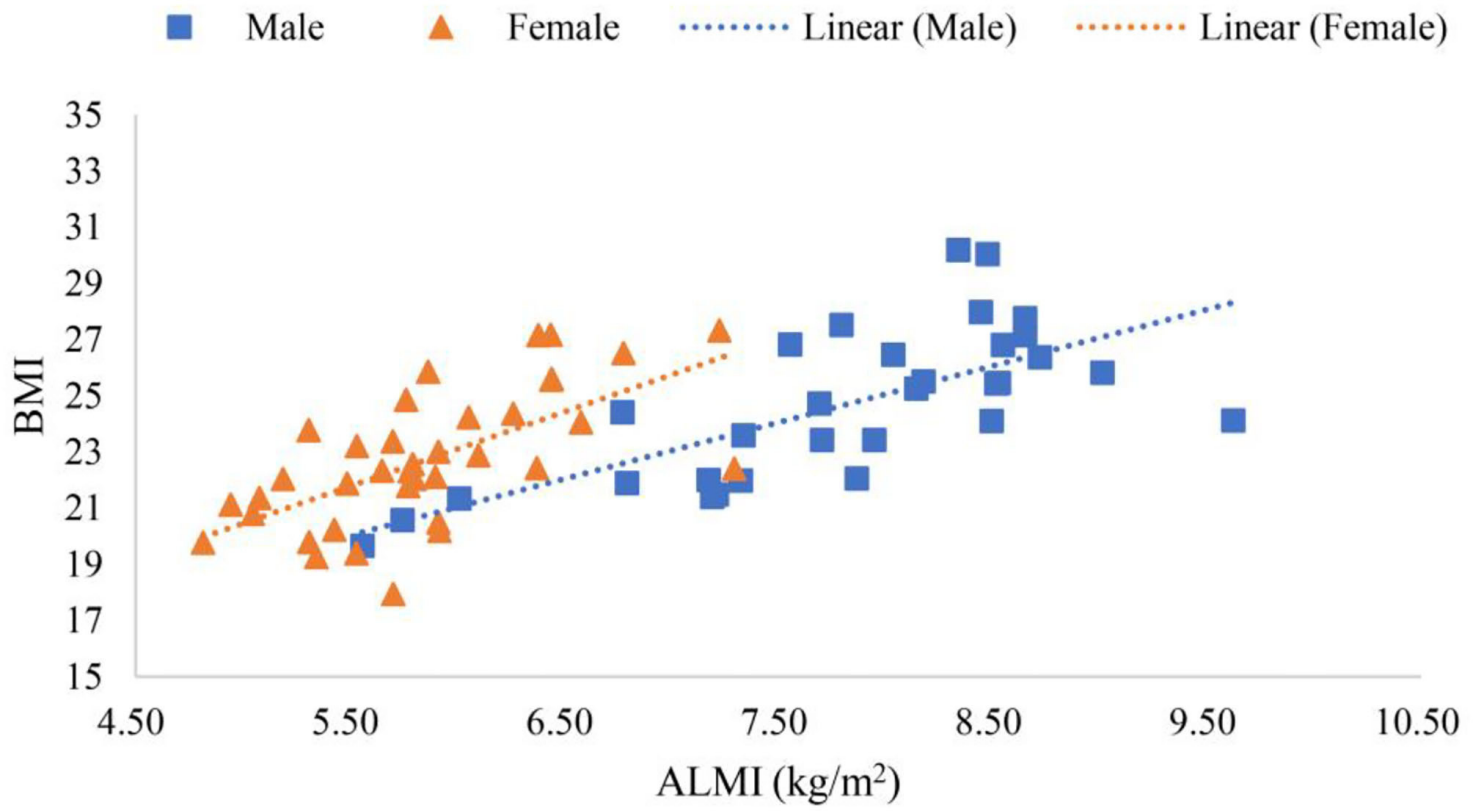
Scatter plots of participants' BMI and ALMI.

**Figure 7:**
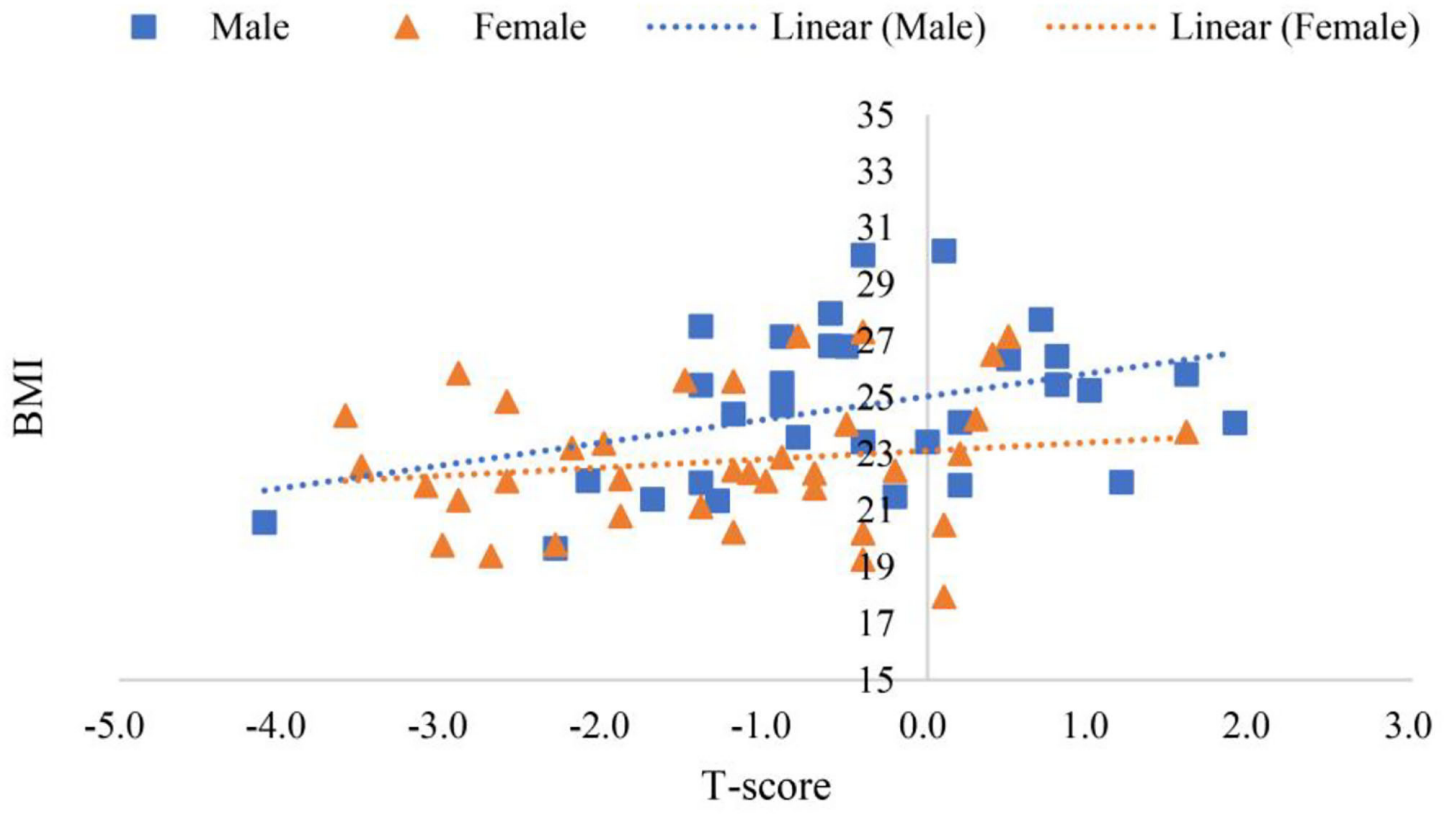
Scatter plots of participants' BMI and *T*-score.

**Figure 8:**
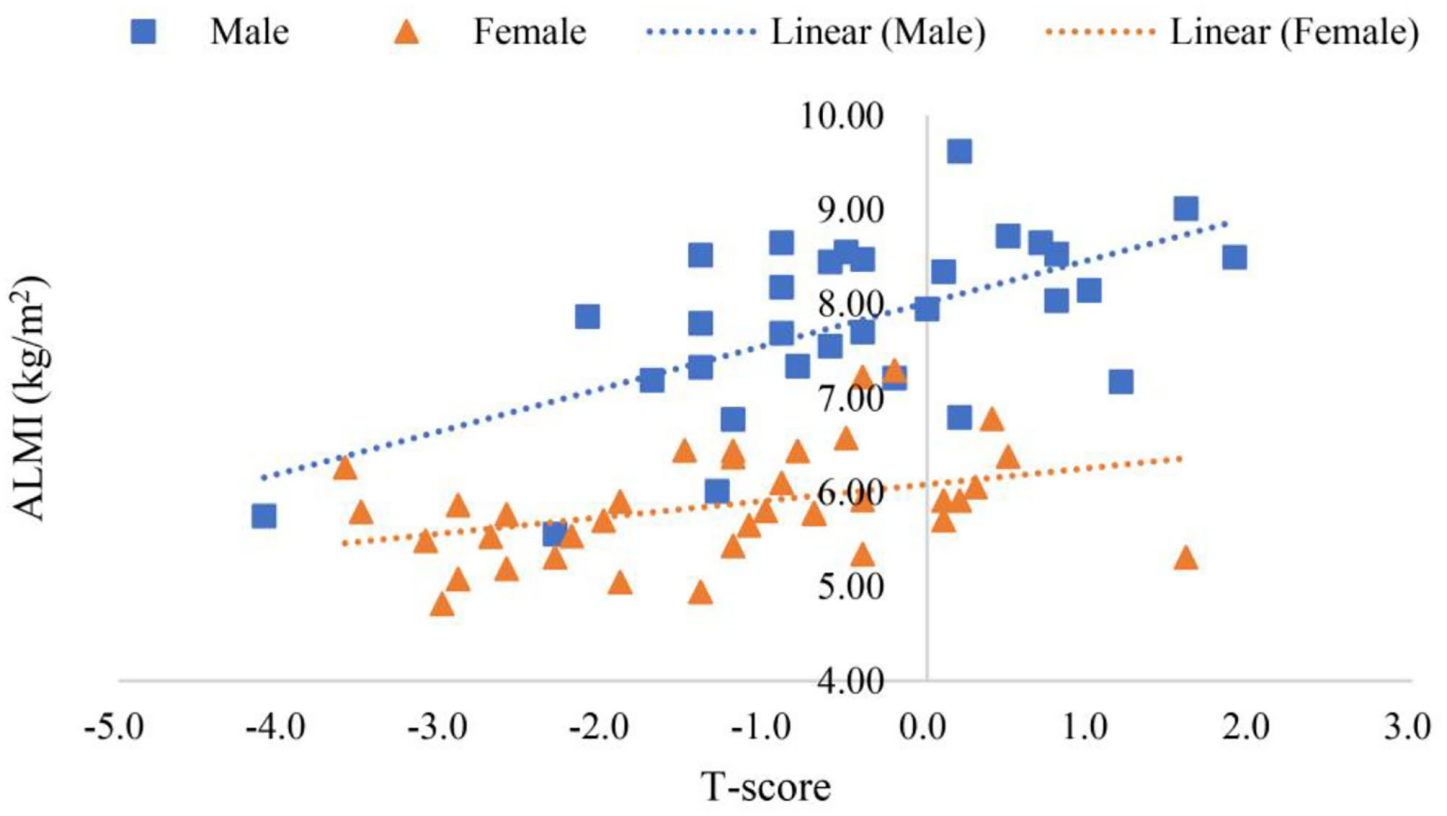
Scatter plots of participants' ALMI and *T*-score.

Scanning reports derived from Hologic QDR-4500A were presented in six scatter plot figures—participants' age and ALMI, age and BMI, age and *T*-score, BMI and ALMI, BMI and *T*-score, and ALMI and *T*-score, as shown in Figures 3–**8**. In addition, linear regression equations were given.

The relationship between gender, age, and ALMI is shown in Figure 3, where *y* (*female*) = −0.0068*x* + 6.3098 (*p* < 0.01), a linear equation of scatter plot of the female's age and ALMI, with the correlation coefficient of 0.06, suggesting a very weak correlation between conditions. Furthermore, *y* (*male*) = −0.0323*x* + 9.9173 (*p* < 0.01) was the linear equation of scatter plot of the male's age and ALMI, with the correlation coefficient of 0.17, suggesting a weak correlation.

The relationship among gender, age, and ALMI is shown in Figure 4, where *y* (*female*) = −0.0084*x* + 23.318 (*p* < 0.01) was a linear equation of scatter plot of the female's age and BMI, with the correlation coefficient of 0.05, suggesting a very weak association. *In addition, y* (*male*) = −0.0096*x* + 24.045 (*p* < 0.01) was a linear equation of scatter plot of the male's age and BMI, with the correlation coefficient of 0.5, also suggesting a moderate correlation.

The relationship between among gender, age, and *T*-score is shown in Figure 5, where *y* (*female*) = 0.0073*x* 1.759 (*p* < 0.01) was a linear equation of scatter plot of the female's age and *T*-score, with the correlation coefficient of 0.03, showing a very weak association. Whereas, *y* (*male*) = 0.0308*x* 2.4408 (*p* < 0.01) was a linear equation of scatter plot of male age and *T*-score, with the correlation coefficient of 0.13, suggesting a very weak correlation.

The relationship between BMI and ALMI in female and male is shown in Figure 6, where *y* (*female*) = 2.5775*x* + 7.6501 (*p* < 0.001) was a linear equation of scatter plot of the female's BMI and ALMI, with the correlation coefficient of 0.62, suggesting a strong correlation. Whereas, *y* (*male*) = 1.9554*x* + 9.4156 (*p* < 0.001) was a linear equation of scatter plot of the male's BMI and ALMI, with the correlation coefficient of 0.69, also suggesting a strong correlation.

The relationship between BMI and *T*-score in female and male is shown in Figure 7, where *y* (*female*) = 0.0218*x* + 22.797 (*p* < 0.01) was a linear equation of scatter plot of the female's BMI and *T*-score, with the correlation coefficient of 0.01, suggesting a very weak association; *y* (*male*) = 0.7615*x* + 24.997 (*p* < 0.01) was a linear equation of scatter plot of the male's BMI and *T*-score, with the correlation coefficient of 0.35, suggesting a weak correlation.

The relationship between ALMI and *T*-score in female and male is shown in **Figure 8**. In **Figure 8**, where *y* (*female*) = 0.1735*x* + 6.0839 (*p* < 0.01) was a linear equation of scatter plot of the female's ALMI and *T-*score, with the correlation coefficient of 0.38, suggesting a weak correlation; *y* (*male*) = 0.4529*x* + 8.0133 (*p* < 0.001) was a linear equation of scatter plot of the male's ALMI and *T*-score, with the correlation coefficient of 0.60, suggesting a moderate correlation.

### Comparisons Between Groups

Comparisons between the sarcopenia and normal groups are presented below and more details are shown in Tables 2, 3.

**Table 2:** Basic gait parameters of the participants.

		**Sarcopenia (*n* = 24)**	**Normal (*n* = 44)**
Step length, cm	Left	59.6 ± 4.96^♦^	64.04 ± 7.52^♦^
	Right	59.78 ± 4.36^♦^	63.96 ± 7.15^♦^
Step time, sec	Left	0.52 ± 0.03	0.53 ± 0.05
	Right	0.52 ± 0.04	0.53 ± 0.05
Stance phase, %	Left	62.74 ± 1.43	62.44 ± 1.70
	Right	63.03 ± 1.62	62.98 ± 1.72
Swing phase, %	Left	37.26 ± 1.43	37.56 ± 1.70
	Right	36.97 ± 1.62	37.02 ± 1.72
Double stance phase, %		25.61 ± 2.83	25.13 ± 3.07
Stride length, cm		119.39 ± 9.15^♦^	128.01 ± 14.46^♦^
Stride time, sec		1.05 ± 0.08	1.06 ± 0.09
Cadence, steps/min		115.10 ± 7.81	113.11 ± 9.66
Velocity, km/h		1.14 ± 0.14	1.21 ± 0.18

♦ *p < 0.01; Paired samples t-test (two-tailed distribution) is used for the gait parameters of participants in both groups*.

**Table 3:** Butterfly parameters of the participants.

		**Sarcopenia (*n* = 24)**	**Normal (*n* = 44)**
Length of gait line, mm	Left	195.17 ± 15.10^♢^	205.28 ± 17.29^♢^
	Right	193.53 ± 15.60^♦^	205.31 ± 18.15^♦^
Single support line. mm	Left	111.08 ± 7.12^♦^	117.21 ± 11.45^♦^
	Right	110.11 ± 12.19^♢^	117.21 ± 11.24^♢^
Anterior/posterior position, mm		74.24 ± 63.68	97.64 ± 64.24
Anterior/posterior position variability, mm		2.98 ± 2.98	2.43 ± 1.41
Lateral symmetry, mm		0.03 ± 4.71	−0.71 ± 3.83
Lateral variability, mm		3.59 ± 2.84	2.6 ± 1.33
Max gait line velocity, cm/sec		153.64 ± 93.07	143.87 ± 114.64

♦ *p < 0.01*,

♢ *p < 0.05; Paired samples t-test (two-tailed distribution) is used for the gait parameters of participants in both groups*.

Table 2 demonstrates extremely significant differences in both sides' step length and stride length between the sarcopenia group and the normal group (*p* < 0.01). The mean cadence of the sarcopenia group was higher than that of the normal group, and the mean step speed is smaller than that of the normal group, but there were no significant differences between them.

Table 3 demonstrates extremely significant differences in the length of right gait line and left single support line between the two groups (*p* < 0.01). Significant differences were found in the length of left gait line and right single support line (*p* < 0.05). The mean values of anterior/posterior position variability, lateral variability, and max gait line velocity of the sarcopenia group were greater than those of the normal group, but without significant difference in all of them.

**Figure 9:**
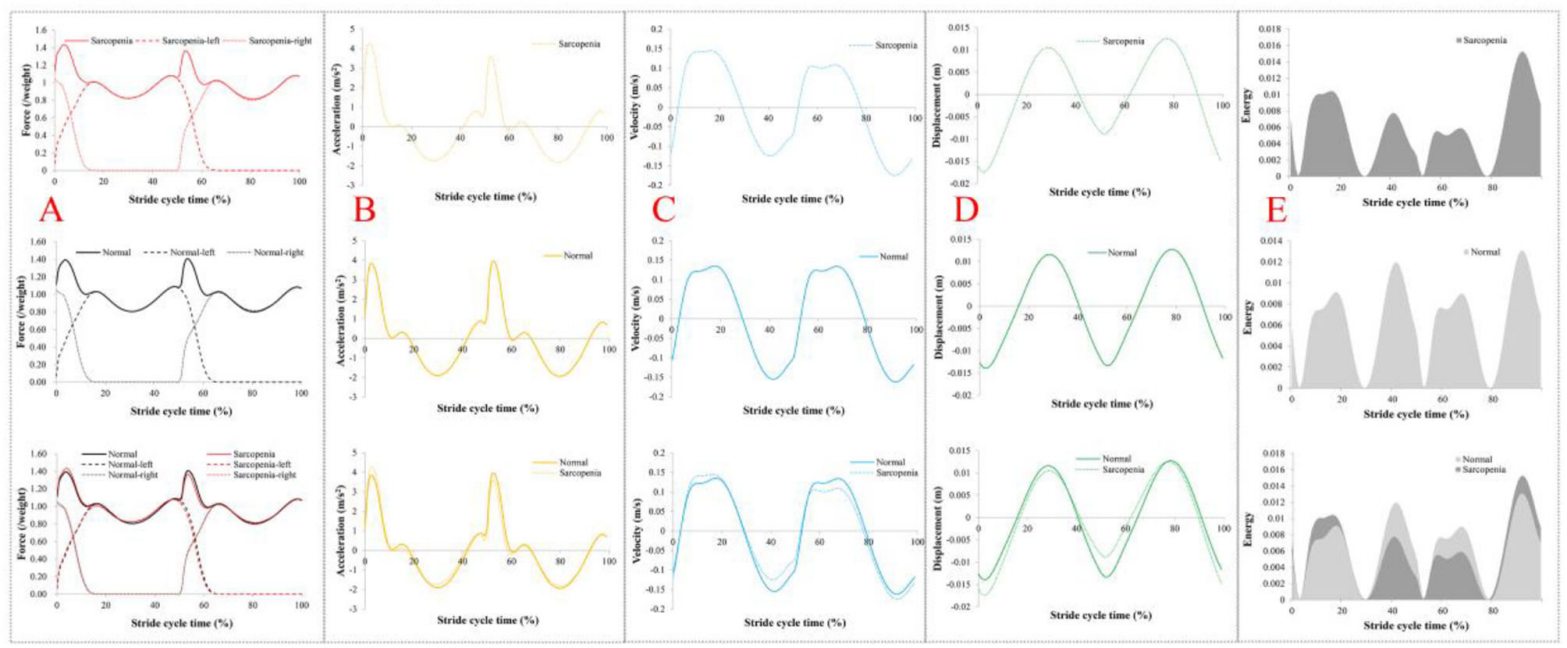
Dynamics results of center of mass (COM). **(A)** The vertical ground reaction force (VGRF) on COM in a stride cycle. **(B)** Vertical acceleration of COM in a stride cycle. **(C)** Vertical velocity of the COM in the stride cycle. **(D)** Vertical displacement of COM in a stride cycle. **(E)** The vertical dynamic energy of COM in a stride cycle. In **(A)**, the dashed line is VGRF on both sides, and the solid line is the resultant force. In **(B–D)**, dashed lines represent the sarcopenia group and solid lines the normal group. In **(E)**, the dark color is sarcopenia and the light color is normal.

Equations (1)–(9) were established to calculate both groups' VGRF, acceleration, velocity, displacement, and dynamic energy of COM, respectively, and the results are shown in **Figure 9**, where the variation trend of VGRF with standardized weight of both groups was similar in a stride cycle. However, the peak value of the left resultant VGRF in the sarcopenia group was greater than that of the normal group, while the peak value of the right resultant VGRF was smaller than that of the normal group. In both groups, the resultant VGRF peaked at 1.4 times of their body weight. The other dynamic quantities of the COM conformed to these characteristics.

## Discussion

This study aimed to explore body composition and gait characteristics in those with sarcopenia in relation to healthy controls as well as the relationship among body composition, gait parameters, and sarcopenia to shed some light on the prevention of falls among the elderly. Importantly, in this study, we calculated gait parameters and COM dynamics between the two groups by providing correlations between body composition and gender and age. We found that the elderly with sarcopenia have abnormal gait parameters, which may be useful to predict and assess the risk of sarcopenia using a simple gait test.

Research has shown that the limb muscles of the elderly are reduced in the cross-sectional area by 25–35% compared with those of the young (Lexell, 1995; Jeon et al., 2021). Sarcopenia occurs with aging, and it is a major factor for frailty (Rolland et al., 2008), mainly due to age-related declines in muscle strength and function, that affects postural reflexes (Landi et al., 2012). Figure 3 shows that ALMI of the female was not correlated with age, while that in men, it was weakly correlated. What was notable was the weak correlation among elderly men. For example, Supplementary Table 4 shows 13 participants with sarcopenia before the age of 65 years, including 10 women and 3 men. Among participants over 65 years old, 11 suffered from sarcopenia. Lifestyle habits, exercise, diet (Fiatarone et al., 1994), and sleep may reduce the incidence of sarcopenia (Piovezan et al., 2015). For instance, resistance exercise was reported to increase muscle mass and strength even in the very elderly (Fiatarone et al., 1994; Hurst et al., 2022).

Body mass index is the gold standard for diagnosing obesity (Romero-Corral et al., 2008; Liu et al., 2022), which is considered a metabolic disease (Pedersen and Saltin, 2015; Korac et al., 2021), and associated with many other comorbidities (Afolabi et al., 2020). For example, obese patients often have hypertension or symptomatic ischemic cardiovascular disease (Johns et al., 2014; Valensi et al., 2021). However, a U-shaped association has been reported between BMI and mortality rate (Allison et al., 2008; Childers and Allison, 2010). Figure 4 showed that age in the elderly was not correlated with BMI, which may be explained by the “obesity paradox” (Childers and Allison, 2010). In Table 1, the BMI of the sarcopenia group was 21.48 ± 1.73 and that of the normal group was 24.77 ± 2.43, and significant difference (*p* < 0.001) was found. It may provide evidence to the fact that people with moderate BMI tend to live longer than those with a higher or lower BMI (Childers and Allison, 2010).

Figure 5 shows that the age of the female was not correlated to *T*-score while that of the male was weakly correlated. Using only a certain part of bone mineral density (g/cm^2^) to diagnose osteoporosis may lead to misdiagnose (Binkley et al., 2005). In this study, “e Whole Body” scanning mode and “Auto Whole Body” analysis were used to avoid measurement errors when selecting measuring body part (Bazzocchi et al., 2016). Results from Figure 5 revealed that the decreasing trend of bone mineral density with age was indisputable (Pocock et al., 1989; O'Gorman et al., 2022). However, lifestyle and other factors may slow down the possibility of suffering from osteoporosis due to aging (Zhu and Prince, 2015). However, in Supplementary Tables 4, 5, among the 41 participants younger than 65 years old, the number of people with low *T*-score (*T*-score < −1) reached 18 (Kanis et al., 1994), which should be attended. This confirms the relationship between “functional vs. chronological age” and disease again (Soto-Perez-de-Celis et al., 2018).

Figure 6 shows that BMI was positively correlated with the ALMI in all participants, though moderately correlated, with correlation coefficients greater than 0.6 for both women and men. The U-shaped association between BMI and mortality rate (Allison et al., 2008; Childers and Allison, 2010) highlighted the importance of moderate BMI for the elderly. Table 1 shows that, within the range of normal BMI values, it was wise to choose the Highest Normal, because Figure 6 reflected the positive correlation between BMI and ALMI, indicating that maintaining a BMI of Highest Normal was significantly correlated with the absence of sarcopenia (*p* < 0.001).

Figure 7 shows that BMI of the female was not correlated with *T*-score while that of the male was moderately correlated. Figure 5 shows that the female's *T*-score was not correlated with age nor BMI, while that of the male was positively correlated with age and BMI. Though the correlation coefficient was low, the gender difference was significant. The reason remains to be further investigated (Greco et al., 2010; Kim et al., 2017; Wung et al., 2021).

Sarcopenia and osteoporosis are two diseases that require further exploration for evidence of biochemical and molecular interactions between the two (Reginster et al., 2016). Diagnostic standards for sarcopenia were controversial (Edwards et al., 2015; Landi et al., 2018), but the ALMI was an indicator for diagnosing sarcopenia, and *T*-score an indicator for diagnosing osteoporosis. According to the diagnostic standard of *T*-score (Kanis et al., 1994; El Maghraoui and Roux, 2008), in the morbidity rate of osteopenia and osteoporosis in sarcopenia was 75.00%, while the morbidity rate in the normal group was 25.00% as shown in Supplementary Tables 4, 5. The morbidity rate of osteoporosis in sarcopenia was 33.33%, while that in the normal group was 4.55%. The moderate positive correlation between ALMI and *T*-score of all participants in Figure 8 was consistent with the connotation of the difference in the incidence of osteoporosis between both groups presented in Supplementary Tables 4, 5.

Table 2 shows that no statistical difference was found in parameters, such as stride frequency and stride time between both groups (*p* > 0.05), and there were significant differences in bilateral stride length and step lengths (*p* < 0.01). Though considerable debate about the value of step velocity was ongoing (Kim et al., 2012; Liu et al., 2013; Tanimoto et al., 2014), consensus had been reached, i.e., slow step velocity was associated with the incidence of sarcopenia. Table 2 confirms the differences in step velocity in patients with sarcopenia—the incidence of sarcopenia was significantly increased when bilateral stride length reduced, which was the main cause of the decreased step velocity (Kim et al., 2021).

The butterfly parameter assessment has been used in the assessment of the patients with acute stroke (Jin, 2010). Butterfly parameters in Table 3 shows significant differences between the sarcopenic group and the normal group in both the bilateral length of the gait line and the single support line. The length of the gait line represented the trajectory of center of pressure during the interaction between the foot and the supporting surface (Lugade and Kaufman, 2014). While walking, the support phase includes single support and double support phase. The ratio of the support phase was correlated to the walking speed, so the faster the walking speed, the smaller the support phase ratio (Fan et al., 2016). No statistical difference between the support phase and the swing phase was found between both the groups in Table 2, and the shorter the single support line in Table 3 the longer the double support phase, which was normal for sarcopenia. In addition, aging is accompanied by the decline of the function of the first metatarsophalangeal joint (Fan et al., 2016), and the shorter length of gait line in sarcopenia in Table 3 offered evidence for the decline of the first metatarsophalangeal joint during the propulsive phase.

The COM dynamics method is used to diagnose diseases of the musculoskeletal system of the lower limb by the changing law of force, acceleration, velocity, and displacement of the COM during walking (Fan et al., 2009, 2016). Figure 7 shows that sarcopenia is neither an asymmetric musculoskeletal disease caused by sports injury or a motor nerve-induced musculoskeletal condition (Fan et al., 2009), but a disease of decreased appendicular muscle mass (Ribeiro and Kehayias, 2014). It can be concluded that the method of assessing sarcopenia by ALMI from a DXA scanner for the assessment of sarcopenia is reliable. Furthermore, considering the radiation of X-ray and the cost of the DXA, a simple gait test for screening sarcopenia offers an attractive new method.

From Tables 2, 3, significant differences were found in step length, stride length, length of gait line, and single support line between the sarcopenia group and the normal group, but no significant difference was found in velocity or cadence, suggesting that sarcopenia was related to the reduction of muscle mass. In addition, Figure 9 shows that in the load response phase, the VGRF peak values from the left and right side of the sarcopenia group were 1.43 and 1.36 times of their body weight, respectively, while those of the normal group were 1.39 and 1.40 times, respectively, suggesting that no significant difference was found in relative muscle strength between these two groups. In Table 1, however, significant difference was found in BMI between these two groups, and BMI of the normal group (115.33%) was greater than that of the sarcopenia group, suggesting that significant difference was found in the absolute muscle strength. Observations of gait parameters and mechanical parameters showed that sarcopenia was a disease mainly caused by decreased muscle mass.

The reduced muscle mass led to the shorter step length, length of gait line, and single support line, which means a change in the gait pattern of the sarcopenia elderly. An increase in cadence and decrease in stride length in the elderly would not cause instability (Fan et al., 2016). However, the cadence of the sarcopenia group was very similar to that of the normal group, and the absolute muscle strength of the sarcopenia group decreased, suggesting decreased stability of body control and increased risk of falling. This study offers a preliminary screening for sarcopenia by a gait test and BMI calculation, speeding up screening and greatly reducing the cost of testing. In addition, the mortality rate of the elderly with slow walking speed was higher than that of the elderly with fast walking speed. The general manifestation of the elderly with slow walking speed included: the older the age, the higher their BMI, depressive mood, and less daily exercise (Harwood and Conroy, 2009; Studenski et al., 2011), consistent with the results of this study.

The study's limitations included the following: (1) elderly male generally reported faster velocity and longer stride length and lower cadence than those of the female, suggesting that there may be gender differences in gait in elderly men and women (Winter et al., 1990; Román et al., 2014). Due to the limited number of community dwellers' screening, both male and female participants were included in the comparative analysis of this study, so future analyses should be conducted with a larger sample size with separated gender; (2) participants were differentiated only by ALMI, calculated from ALM obtained from DXA scans, and no other functional tests were performed to functionally diagnose sarcopenia. A longitudinal study to correlate changes in body composition with gait stability during aging can be an important future research topic.

## Conclusion

This study examined the correlations between body composition and gender and age in the elderly with sarcopenia by ALMI screening as well as differences in gait parameters between healthy controls and patients with sarcopenia. For the elderly, age did not largely affect ALMI, BMI, or *T*-score, but BMI and ALMI were strongly correlated. Significant differences were found in certain gait parameters between the elderly with sarcopenia and normal elderly. The dynamics of COM based on the gait forward dynamics method showed that sarcopenia is a disease mainly caused by decreased muscle mass. When abnormities were found in step length, stride length, length of gait line, or single support line, a DXA scan can then be performed. This may shed some light on the prevention of falls among the elderly.

## Data Availability Statement

The raw data supporting the conclusions of this article will be made available by the authors, without undue reservation.

## Ethics Statement

The studies involving human participants were reviewed and approved by Ethics Committee of Fujian Normal University. The patients/participants provided their written informed consent to participate in this study.

## Author Contributions

YiF conceived and designed the study. YuF, BZ, and GH performed the experiments and collected patients' data. YuF, BZ, GH, GZ, and ZD performed the statistical analysis. YiF and YuF wrote the first draft of the manuscript. ZL and JS reviewed and edited the manuscript. All authors contributed to the manuscript revision and read and approved the submitted version.

## Funding

This study was supported by the National Natural Science Foundation of China (Grant No. 11972119) and the Natural Science Foundation of Fujian Province (Grant No. 2019J01429). The funders played no role in the study design, data collection, analyses, interpretation, manuscript writing, or submission.

## Conflict of Interest

The authors declare that the research was conducted in the absence of any commercial or financial relationships that could be construed as a potential conflict of interest.

## Publisher's Note

All claims expressed in this article are solely those of the authors and do not necessarily represent those of their affiliated organizations, or those of the publisher, the editors and the reviewers. Any product that may be evaluated in this article, or claim that may be made by its manufacturer, is not guaranteed or endorsed by the publisher.

## Abbreviations

ALM: Appendicular lean mass
ALMI: appendicular lean mass index
BMI: Body mass index
COM: Center of mass
DXA: Dual-energy X-ray absorptiometry
VGRF: vertical ground reaction force
GRF: ground reaction force.
