# Supplementary material for: Sarcopenia: Body Composition and Gait Analysis
Source: Front Aging Neurosci. 2022 Jul 13;14:909551. doi: 10.3389/fnagi.2022.909551 (PMC9326397; doi:10.3389/fnagi.2022.909551)
Supplement: Supplementary file 1 [file Data_Sheet_1.PDF]

Supplementary Material

Supplementary Table 1 – 3: an application of the equations.

Force measurement data and high-speed video data of sprinter's running were retrieved from Locomotor Performance Laboratory of Southern Methodist University, USA (https://www.smu.edu/simmons/Research/Locomotor-Performance-Laboratory). We have obtained email permission from Dr. Peter Weyand, the director of laboratory, to use the video clip.

With the video analysis method, we recorded the ground reaction force of Jeremy Wariner during a running stride cycle. Ground reaction force was normalized by self-weight, and stride cycle normalized by percentage. Results were shown in Supplementary Table 1-3:

Supplementary Table 1. Calculation of vertical forward dynamic center of mass

Time%	$F_1^z(t)$	$F_2^z(t)$	$F_z(t)$	$a_z(t)$	$v_z(t)$	$s_z(t)$	$E_z(t)$
0	0	0	0	-9.8	-0.707	-0.005	0.250
1	0.190	0	0.190	-7.934	-0.746	-0.008	0.278
2	0.694	0	0.694	-2.998	-0.761	-0.012	0.290
3	1.453	0	1.453	4.437	-0.739	-0.016	0.273
4	2.051	0	2.051	10.297	-0.688	-0.019	0.237
5	2.548	0	2.548	15.171	-0.612	-0.022	0.187
6	2.945	0	2.945	19.061	-0.517	-0.025	0.134
7	3.241	0	3.241	21.965	-0.408	-0.027	0.083
8	3.437	0	3.437	23.884	-0.289	-0.028	0.042
9	3.532	0	3.532	24.818	-0.165	-0.029	0.014
10	3.526	0	3.526	24.752	-0.042	-0.029	0.001
11	3.506	0	3.506	24.554	0.081	-0.029	0.003
12	3.456	0	3.456	24.065	0.201	-0.028	0.020
13	3.355	0	3.355	23.077	0.316	-0.026	0.050
14	3.189	0	3.189	21.455	0.423	-0.024	0.089
15	2.952	0	2.952	19.133	0.518	-0.022	0.134
16	2.644	0	2.644	16.112	0.598	-0.019	0.179
17	2.272	0	2.272	12.468	0.660	-0.015	0.218
18	1.851	0	1.851	8.343	0.702	-0.012	0.246
19	1.403	0	1.403	3.949	0.722	-0.008	0.260
20	0.956	0	0.956	-0.429	0.719	-0.005	0.259
21	0.547	0	0.547	-4.440	0.697	-0.001	0.243
22	0.218	0	0.218	-7.660	0.659	0.002	0.217
23	0.021	0	0.021	-9.596	0.611	0.005	0.187
24	0.012	0	0.012	-9.687	0.563	0.008	0.158
25	0	0	0	-9.8	0.514	0.010	0.132
26	0	0	0	-9.8	0.465	0.013	0.108
27	0	0	0	-9.8	0.417	0.015	0.087
28	0	0	0	-9.8	0.368	0.017	0.068
29	0	0	0	-9.8	0.319	0.018	0.051
30	0	0	0	-9.8	0.270	0.020	0.036
31	0	0	0	-9.8	0.221	0.021	0.024
32	0	0	0	-9.8	0.172	0.022	0.015
33	0	0	0	-9.8	0.124	0.022	0.008
34	0	0	0	-9.8	0.075	0.023	0.003
35	0	0	0	-9.8	0.026	0.023	0
36	0	0	0	-9.8	-0.023	0.023	0
37	0	0	0	-9.8	-0.072	0.022	0.003
38	0	0	0	-9.8	-0.121	0.022	0.007
39	0	0	0	-9.8	-0.170	0.021	0.014

40	0	0	0	-9.8	-0.218	0.020	0.024
41	0	0	0	-9.8	-0.267	0.018	0.036
42	0	0	0	-9.8	-0.316	0.017	0.050
43	0	0	0	-9.8	-0.365	0.015	0.067
44	0	0	0	-9.8	-0.414	0.013	0.086
45	0	0	0	-9.8	-0.463	0.011	0.107
46	0	0	0	-9.8	-0.511	0.008	0.131
47	0	0	0	-9.8	-0.560	0.005	0.157
48	0	0	0	-9.8	-0.609	0.002	0.185
49	0	0	0	-9.8	-0.658	-0.001	0.216
50	0	0	0	-9.8	-0.707	-0.005	0.250
51	0	0.190	0.190	-7.934	-0.746	-0.008	0.278
52	0	0.694	0.694	-2.998	-0.761	-0.012	0.290
53	0	1.453	1.453	4.437	-0.739	-0.016	0.273
54	0	2.051	2.051	10.297	-0.688	-0.019	0.237
55	0	2.548	2.548	15.171	-0.612	-0.022	0.187
56	0	2.945	2.945	19.061	-0.517	-0.025	0.134
57	0	3.241	3.241	21.965	-0.408	-0.027	0.083
58	0	3.437	3.437	23.884	-0.289	-0.028	0.042
59	0	3.532	3.532	24.818	-0.165	-0.029	0.014
60	0	3.526	3.526	24.752	-0.042	-0.029	0.001
61	0	3.506	3.506	24.554	0.081	-0.029	0.003
62	0	3.456	3.456	24.065	0.201	-0.028	0.020
63	0	3.355	3.355	23.077	0.316	-0.026	0.050
64	0	3.189	3.189	21.455	0.423	-0.024	0.089
65	0	2.952	2.952	19.133	0.518	-0.022	0.134
66	0	2.644	2.644	16.112	0.598	-0.019	0.179
67	0	2.272	2.272	12.468	0.660	-0.015	0.218
68	0	1.851	1.851	8.343	0.702	-0.012	0.246
69	0	1.403	1.403	3.949	0.722	-0.008	0.260
70	0	0.956	0.956	-0.429	0.719	-0.005	0.259
71	0	0.547	0.547	-4.440	0.697	-0.001	0.243
72	0	0.218	0.218	-7.660	0.659	0.002	0.217
73	0	0.021	0.021	-9.596	0.611	0.005	0.187
74	0	0.012	0.012	-9.687	0.563	0.008	0.158
75	0	0	0	-9.8	0.514	0.010	0.132
76	0	0	0	-9.8	0.465	0.013	0.108
77	0	0	0	-9.8	0.417	0.015	0.087
78	0	0	0	-9.8	0.368	0.017	0.068
79	0	0	0	-9.8	0.319	0.018	0.051
80	0	0	0	-9.8	0.270	0.020	0.036
81	0	0	0	-9.8	0.221	0.021	0.024
82	0	0	0	-9.8	0.172	0.022	0.015
83	0	0	0	-9.8	0.124	0.022	0.008
84	0	0	0	-9.8	0.075	0.023	0.003
85	0	0	0	-9.8	0.026	0.023	0
86	0	0	0	-9.8	-0.023	0.023	0
87	0	0	0	-9.8	-0.072	0.022	0.003
88	0	0	0	-9.8	-0.121	0.022	0.007
89	0	0	0	-9.8	-0.170	0.021	0.014
90	0	0	0	-9.8	-0.218	0.020	0.024
91	0	0	0	-9.8	-0.267	0.018	0.036
92	0	0	0	-9.8	-0.316	0.017	0.050
93	0	0	0	-9.8	-0.365	0.015	0.067
94	0	0	0	-9.8	-0.414	0.013	0.086
95	0	0	0	-9.8	-0.463	0.011	0.107
96	0	0	0	-9.8	-0.511	0.008	0.131
97	0	0	0	-9.8	-0.560	0.005	0.157
98	0	0	0	-9.8	-0.609	0.002	0.185
99	0	0	0	-9.8	-0.658	-0.001	0.216

Supplementary Table 2. Calculation of medio-lateral forward dynamic center of mass

Time%	$F_1^y(t)$	$F_2^y(t)$	$F_y(t)$	$a_y(t)$	$v_y(t)$	$s_y(t)$	$E_y(t)$
0	0	0	0	0	-0.061	-0.004	0.002
1	-0.024	0	-0.024	-0.232	-0.062	-0.004	0.002
2	-0.049	0	-0.049	-0.481	-0.065	-0.004	0.002
3	-0.075	0	-0.075	-0.737	-0.068	-0.005	0.002
4	-0.077	0	-0.077	-0.750	-0.072	-0.005	0.003
5	-0.019	0	-0.019	-0.182	-0.073	-0.006	0.003
6	0.065	0	0.065	0.637	-0.070	-0.006	0.002
7	0.150	0	0.150	1.471	-0.063	-0.006	0.002
8	0.212	0	0.212	2.080	-0.052	-0.006	0.001
9	0.297	0	0.297	2.908	-0.038	-0.007	0.001
10	0.312	0	0.312	3.055	-0.022	-0.007	0
11	0.285	0	0.285	2.796	-0.009	-0.007	0
12	0.244	0	0.244	2.387	0.003	-0.007	0
13	0.203	0	0.203	1.990	0.013	-0.007	0
14	0.172	0	0.172	1.686	0.022	-0.007	0
15	0.153	0	0.153	1.496	0.029	-0.006	0
16	0.143	0	0.143	1.398	0.036	-0.006	0.001
17	0.137	0	0.137	1.339	0.043	-0.006	0.001
18	0.128	0	0.128	1.259	0.049	-0.006	0.001
19	0.113	0	0.113	1.104	0.055	-0.006	0.001
20	0.086	0	0.086	0.846	0.059	-0.005	0.002
21	0.051	0	0.051	0.495	0.061	-0.005	0.002
22	0.013	0	0.013	0.123	0.062	-0.005	0.002
23	-0.013	0	-0.013	-0.124	0.061	-0.004	0.002
24	-0.001	0	-0.001	-0.008	0.061	-0.004	0.002
25	0	0	0	0	0.061	-0.004	0.002
26	0	0	0	0	0.061	-0.003	0.002
27	0	0	0	0	0.061	-0.003	0.002
28	0	0	0	0	0.061	-0.003	0.002
29	0	0	0	0	0.061	-0.003	0.002
30	0	0	0	0	0.061	-0.002	0.002
31	0	0	0	0	0.061	-0.002	0.002
32	0	0	0	0	0.061	-0.002	0.002
33	0	0	0	0	0.061	-0.001	0.002
34	0	0	0	0	0.061	-0.001	0.002
35	0	0	0	0	0.061	-0.001	0.002
36	0	0	0	0	0.061	0	0.002
37	0	0	0	0	0.061	0	0.002
38	0	0	0	0	0.061	0	0.002
39	0	0	0	0	0.061	0.001	0.002
40	0	0	0	0	0.061	0.001	0.002
41	0	0	0	0	0.061	0.001	0.002
42	0	0	0	0	0.061	0.001	0.002
43	0	0	0	0	0.061	0.002	0.002
44	0	0	0	0	0.061	0.002	0.002
45	0	0	0	0	0.061	0.002	0.002
46	0	0	0	0	0.061	0.003	0.002
47	0	0	0	0	0.061	0.003	0.002
48	0	0	0	0	0.061	0.003	0.002
49	0	0	0	0	0.061	0.004	0.002
50	0	0	0	0	0.061	0.004	0.002
51	0	0.024	0.024	0.232	0.062	0.004	0.002
52	0	0.049	0.049	0.481	0.065	0.004	0.002
53	0	0.075	0.075	0.737	0.068	0.005	0.002
54	0	0.077	0.077	0.750	0.072	0.005	0.003
55	0	0.019	0.019	0.182	0.073	0.006	0.003
56	0	-0.065	-0.065	-0.637	0.070	0.006	0.002
57	0	-0.150	-0.150	-1.471	0.063	0.006	0.002

58	0	-0.212	-0.212	-2.080	0.052	0.006	0.001
59	0	-0.297	-0.297	-2.908	0.038	0.007	0.001
60	0	-0.312	-0.312	-3.055	0.022	0.007	0
61	0	-0.285	-0.285	-2.796	0.009	0.007	0
62	0	-0.244	-0.244	-2.387	-0.003	0.007	0
63	0	-0.203	-0.203	-1.990	-0.013	0.007	0
64	0	-0.172	-0.172	-1.686	-0.022	0.007	0
65	0	-0.153	-0.153	-1.496	-0.029	0.006	0
66	0	-0.143	-0.143	-1.398	-0.036	0.006	0.001
67	0	-0.137	-0.137	-1.339	-0.043	0.006	0.001
68	0	-0.128	-0.128	-1.259	-0.049	0.006	0.001
69	0	-0.113	-0.113	-1.104	-0.055	0.006	0.001
70	0	-0.086	-0.086	-0.846	-0.059	0.005	0.002
71	0	-0.051	-0.051	-0.495	-0.061	0.005	0.002
72	0	-0.013	-0.013	-0.123	-0.062	0.005	0.002
73	0	0.013	0.013	0.124	-0.061	0.004	0.002
74	0	0.001	0.001	0.008	-0.061	0.004	0.002
75	0	0	0	0	-0.061	0.004	0.002
76	0	0	0	0	-0.061	0.003	0.002
77	0	0	0	0	-0.061	0.003	0.002
78	0	0	0	0	-0.061	0.003	0.002
79	0	0	0	0	-0.061	0.003	0.002
80	0	0	0	0	-0.061	0.002	0.002
81	0	0	0	0	-0.061	0.002	0.002
82	0	0	0	0	-0.061	0.002	0.002
83	0	0	0	0	-0.061	0.001	0.002
84	0	0	0	0	-0.061	0.001	0.002
85	0	0	0	0	-0.061	0.001	0.002
86	0	0	0	0	-0.061	0	0.002
87	0	0	0	0	-0.061	0	0.002
88	0	0	0	0	-0.061	0	0.002
89	0	0	0	0	-0.061	-0.001	0.002
90	0	0	0	0	-0.061	-0.001	0.002
91	0	0	0	0	-0.061	-0.001	0.002
92	0	0	0	0	-0.061	-0.001	0.002
93	0	0	0	0	-0.061	-0.002	0.002
94	0	0	0	0	-0.061	-0.002	0.002
95	0	0	0	0	-0.061	-0.002	0.002
96	0	0	0	0	-0.061	-0.003	0.002
97	0	0	0	0	-0.061	-0.003	0.002
98	0	0	0	0	-0.061	-0.003	0.002
99	0	0	0	0	-0.061	-0.004	0.002

Supplementary Table 3. Calculation of anterior-posterior forward dynamic center of mass

Time%	$F_1^x(t)$	$F_2^x(t)$	$F_x(t)$	$a_x(t)$	$v_x(t)$	$s_x(t)$	$E_x(t)$
0	0	0	0	0	0.019	0.006	0
1	-0.142	0	-0.142	-1.396	0.012	0.006	0
2	-0.361	0	-0.361	-3.539	-0.005	0.006	0
3	-0.614	0	-0.614	-6.015	-0.035	0.006	0.001
4	-0.650	0	-0.650	-6.366	-0.067	0.006	0.002
5	-0.570	0	-0.570	-5.584	-0.095	0.005	0.005
6	-0.480	0	-0.480	-4.705	-0.118	0.005	0.007
7	-0.412	0	-0.412	-4.034	-0.138	0.004	0.010
8	-0.347	0	-0.347	-3.397	-0.155	0.003	0.012
9	-0.292	0	-0.292	-2.859	-0.170	0.002	0.014
10	-0.223	0	-0.223	-2.184	-0.181	0.001	0.016
11	-0.128	0	-0.128	-1.257	-0.187	0	0.017
12	-0.006	0	-0.006	-0.059	-0.187	-0.001	0.018
13	0.138	0	0.138	1.349	-0.180	-0.001	0.016
14	0.291	0	0.291	2.850	-0.166	-0.002	0.014
15	0.438	0	0.438	4.293	-0.145	-0.003	0.010
16	0.563	0	0.563	5.515	-0.117	-0.004	0.007
17	0.649	0	0.649	6.357	-0.086	-0.004	0.004
18	0.683	0	0.683	6.694	-0.052	-0.004	0.001
19	0.658	0	0.658	6.447	-0.020	-0.004	0
20	0.573	0	0.573	5.612	0.008	-0.004	0
21	0.436	0	0.436	4.276	0.029	-0.004	0
22	0.269	0	0.269	2.641	0.042	-0.004	0.001
23	0.107	0	0.107	1.046	0.048	-0.004	0.001
24	0.002	0	0.002	0.016	0.048	-0.003	0.001
25	0	0	0	0	0.048	-0.003	0.001
26	0	0	0	0	0.048	-0.003	0.001
27	0	0	0	0	0.048	-0.003	0.001
28	0	0	0	0	0.048	-0.003	0.001
29	0	0	0	0	0.048	-0.002	0.001
30	0	0	0	0	0.048	-0.002	0.001
31	0	0	0	0	0.048	-0.002	0.001
32	0	0	0	0	0.048	-0.002	0.001
33	0	0	0	0	0.048	-0.001	0.001
34	0	0	0	0	0.048	-0.001	0.001
35	0	0	0	0	0.048	-0.001	0.001
36	0	0	0	0	0.048	-0.001	0.001
37	0	0	0	0	0.048	0	0.001
38	0	0	0	0	0.048	0	0.001
39	0	0	0	0	0.048	0	0.001
40	0	0	0	0	0.048	0	0.001
41	0	0	0	0	0.048	0.001	0.001
42	0	0	0	0	0.048	0.001	0.001
43	0	0	0	0	0.048	0.001	0.001
44	0	0	0	0	0.048	0.001	0.001
45	0	0	0	0	0.048	0.002	0.001
46	0	0	0	0	0.048	0.002	0.001
47	0	0	0	0	0.048	0.002	0.001
48	0	0	0	0	0.048	0.002	0.001
49	0	0	0	0	0.048	0.002	0.001
50	0	0	0	0	0.048	0.003	0.001
51	0	-0.142	-0.142	-1.396	0.041	0.003	0.001
52	0	-0.361	-0.361	-3.539	0.023	0.003	0
53	0	-0.614	-0.614	-6.015	-0.007	0.003	0
54	0	-0.650	-0.650	-6.366	-0.039	0.003	0.001
55	0	-0.570	-0.570	-5.584	-0.067	0.002	0.002
56	0	-0.480	-0.480	-4.705	-0.090	0.002	0.004
57	0	-0.412	-0.412	-4.034	-0.110	0.001	0.006

58	0	-0.347	-0.347	-3.397	-0.127	0.001	0.008
59	0	-0.292	-0.292	-2.859	-0.141	0	0.010
60	0	-0.223	-0.223	-2.184	-0.152	-0.001	0.012
61	0	-0.128	-0.128	-1.257	-0.158	-0.001	0.013
62	0	-0.006	-0.006	-0.059	-0.159	-0.002	0.013
63	0	0.138	0.138	1.349	-0.152	-0.003	0.012
64	0	0.291	0.291	2.850	-0.138	-0.004	0.009
65	0	0.438	0.438	4.293	-0.116	-0.004	0.007
66	0	0.563	0.563	5.515	-0.089	-0.005	0.004
67	0	0.649	0.649	6.357	-0.057	-0.005	0.002
68	0	0.683	0.683	6.694	-0.024	-0.005	0
69	0	0.658	0.658	6.447	0.008	-0.005	0
70	0	0.573	0.573	5.612	0.036	-0.005	0.001
71	0	0.436	0.436	4.276	0.058	-0.005	0.002
72	0	0.269	0.269	2.641	0.071	-0.004	0.002
73	0	0.107	0.107	1.046	0.076	-0.004	0.003
74	0	0.002	0.002	0.016	0.076	-0.003	0.003
75	0	0	0	0	0.076	-0.003	0.003
76	0	0	0	0	0.076	-0.003	0.003
77	0	0	0	0	0.076	-0.002	0.003
78	0	0	0	0	0.076	-0.002	0.003
79	0	0	0	0	0.076	-0.002	0.003
80	0	0	0	0	0.076	-0.001	0.003
81	0	0	0	0	0.076	-0.001	0.003
82	0	0	0	0	0.076	0	0.003
83	0	0	0	0	0.076	0	0.003
84	0	0	0	0	0.076	0	0.003
85	0	0	0	0	0.076	0.001	0.003
86	0	0	0	0	0.076	0.001	0.003
87	0	0	0	0	0.076	0.001	0.003
88	0	0	0	0	0.076	0.002	0.003
89	0	0	0	0	0.076	0.002	0.003
90	0	0	0	0	0.076	0.003	0.003
91	0	0	0	0	0.076	0.003	0.003
92	0	0	0	0	0.076	0.003	0.003
93	0	0	0	0	0.076	0.004	0.003
94	0	0	0	0	0.076	0.004	0.003
95	0	0	0	0	0.076	0.004	0.003
96	0	0	0	0	0.076	0.005	0.003
97	0	0	0	0	0.076	0.005	0.003
98	0	0	0	0	0.076	0.006	0.003
99	0	0	0	0	0.076	0.006	0.003

Supplementary Table 4. Basic information of DXA scan reports of the sarcopenia group

Participants	Gender	Age	Height (cm)	Weight (kg)	BMI	ALM (g)	AMI	*T*-score
GZS	Male	62	160.80	63.10	24.40	17509.70	6.77	-1.20
LHL	Male	72	165.00	56.00	20.57	15641.80	5.75	-4.10
LXH	Male	78	170.30	63.50	21.89	19704.70	6.79	0.20
WHL	Male	63	158.50	53.60	21.34	15099.70	6.01	-1.30
ZYH	Male	58	153.00	46.00	19.65	13010.20	5.56	-2.30
FLZ	Female	59	156.30	57.10	23.37	13922.90	5.70	-2.00
GS	Female	71	148.70	43.70	19.76	10646.80	4.82	-3.00
FHY	Female	62	154.40	51.90	21.77	13760.30	5.77	-0.70
HSQ	Female	65	153.00	50.00	21.36	11888.60	5.08	-2.90
HZH	Female	69	162.20	47.20	17.94	15005.00	5.70	0.10
LGF	Female	58	150.70	52.70	23.21	12565.30	5.53	-2.20
LDH	Female	70	149.00	46.90	21.13	10976.20	4.94	-1.40
LL	Female	53	169.00	55.00	19.26	15259.60	5.34	-0.40
LXJ	Female	65	153.60	52.00	22.04	12241.00	5.19	-2.60
LXZ	Female	74	153.00	51.20	21.87	12843.60	5.49	-3.10
WLN	Female	58	149.90	46.70	20.78	11340.30	5.05	-1.90
XXL	Female	57	156.50	54.70	22.33	13839.50	5.65	-1.10
YYB	Female	67	156.10	57.90	23.76	12935.90	5.31	1.60
ZWY	Female	56	152.70	45.20	19.38	12892.30	5.53	-2.70
ZJY	Female	56	165.50	54.20	19.79	14543.40	5.31	-2.30
ZYY	Female	66	157.40	50.10	20.22	13446.60	5.43	-1.20
CHL	Female	67	161.50	58.20	22.31	15073.10	5.78	-0.70
LJF	Female	59	168.00	63.70	22.57	16354.00	5.79	-3.50
ZJJ	Female	59	158.60	62.50	24.85	14493.60	5.76	-2.60

Supplementary Table 5. Basic information of DXA scan reports of the normal group

Participants	Gender	Age	Height (cm)	Weight (kg)	BMI	ALM (g)	AMI	*T*-score
CBC	Male	56	160.90	69.40	26.81	22131.00	8.55	-0.50
CDY	Male	70	166.00	65.00	23.59	20218.10	7.34	-0.80
GJF	Male	60	177.40	73.70	23.42	24241.60	7.70	-0.40
GYC	Male	72	168.20	85.00	30.04	23977.00	8.48	-0.40
HWY	Male	69	167.50	60.00	21.39	20173.30	7.19	-1.70
HYK	Male	72	172.30	81.70	27.52	23136.30	7.79	-1.40
HS	Male	60	167.30	78.30	27.97	23636.40	8.44	-0.60
HSQ	Male	56	169.40	63.30	22.06	22573.80	7.87	-2.10
JPL	Male	62	165.70	64.30	23.42	21816.50	7.95	0.00
LZD	Male	63	163.00	58.40	21.98	19463.30	7.33	-1.40
LMJ	Male	74	165.50	82.70	30.19	22843.80	8.34	0.10
LPL	Male	68	167.00	71.00	25.46	23783.20	8.53	0.80
SYW	Male	61	170.00	73.70	25.50	23630.40	8.18	-0.90
SWY	Male	69	168.00	68.00	24.09	23976.40	8.50	1.90
SW	Male	64	175.00	85.00	27.76	26489.20	8.65	0.70
WK	Male	73	170.70	64.10	22.00	20900.80	7.17	1.20
XGZ	Male	59	171.60	72.80	24.72	22649.20	7.69	-0.90
XP	Male	66	171.10	62.90	21.49	21111.80	7.21	-0.20
XXD	Male	60	167.80	71.60	25.43	23977.70	8.52	-1.40
YDW	Male	64	176.00	80.00	25.83	27914.60	9.01	1.60
YQ	Male	64	162.00	63.30	24.12	25249.90	9.62	0.20
ZCC	Male	63	170.00	73.00	25.26	23532.40	8.14	1.00
ZRY	Male	60	173.20	79.10	26.37	26159.60	8.72	0.50
ZXG	Male	62	171.40	78.80	26.82	22192.70	7.55	-0.60
ZYJ	Male	70	160.90	69.40	26.81	22131.00	8.55	-0.50
ZFC	Male	62	166.00	65.00	23.59	20218.10	7.34	-0.80
CAY	Female	68	177.40	73.70	23.42	24241.60	7.70	-0.40
CJW	Female	68	168.20	85.00	30.04	23977.00	8.48	-0.40
DYZ	Female	54	167.50	60.00	21.39	20173.30	7.19	-1.70
HJF	Female	59	172.30	81.70	27.52	23136.30	7.79	-1.40
FQF	Female	61	167.30	78.30	27.97	23636.40	8.44	-0.60
FYY	Female	70	169.40	63.30	22.06	22573.80	7.87	-2.10
GXZ	Female	67	165.70	64.30	23.42	21816.50	7.95	0.00
HHL	Female	56	163.00	58.40	21.98	19463.30	7.33	-1.40
LBM	Female	73	165.50	82.70	30.19	22843.80	8.34	0.10
LHJ	Female	62	167.00	71.00	25.46	23783.20	8.53	0.80
LJF	Female	56	170.00	73.70	25.50	23630.40	8.18	-0.90
LSY	Female	57	168.00	68.00	24.09	23976.40	8.50	1.90
LXH	Female	61	175.00	85.00	27.76	26489.20	8.65	0.70
LXL	Female	58	170.70	64.10	22.00	20900.80	7.17	1.20
WSL	Female	77	171.60	72.80	24.72	22649.20	7.69	-0.90
YJ	Female	59	171.10	62.90	21.49	21111.80	7.21	-0.20
ZML	Female	75	167.80	71.60	25.43	23977.70	8.52	-1.40
ZWM	Female	64	176.00	80.00	25.83	27914.60	9.01	1.60
